# Carvedilol Ameliorates Experimental Atherosclerosis by Regulating Cholesterol Efflux and Exosome Functions

**DOI:** 10.3390/ijms20205202

**Published:** 2019-10-20

**Authors:** Sy-Jou Chen, Pi-Fen Tsui, Yi-Ping Chuang, Dapi Meng-Lin Chiang, Liv Weichien Chen, Shu-Ting Liu, Feng-Yen Lin, Shih-Ming Huang, Shih-Hua Lin, Wan-Lin Wu, Min-Chien Tsai, Chin-Sheng Lin

**Affiliations:** 1 Department of Emergency Medicine, Tri-Service General Hospital, National Defense Medical Center, Taipei 11490, Taiwan; syjou.chen@gmail.com; 2 Graduate Institute of Injury Prevention and Control, College of Public Health and Nutrition, Taipei Medical University, Taipei 11031, Taiwan; 3 Graduate Institute of Life Sciences, National Defense Medical Center, Taipei 11490, Taiwan; befun1214@gmail.com; 4 Department and Graduate Institute of Microbiology and Immunology, National Defense Medical Center, Taipei 11490, Taiwan; ypchuang@mail.ndmctsgh.edu.tw; 5 Biovesicle Inc., Taipei 11490, Taiwan; dapi_chiang@biovesicle.com; 6 Division of Cardiology, Department of Medicine, Tri-Service General Hospital, National Defense Medical Center, Taipei 11490, Taiwan; mslivcat@gmail.com; 7 Department of Biochemistry, National Defense Medical Center, Taipei 11490, Taiwan; shuting0719@gmail.com (S.-T.L.); shihming@mail.ndmctsgh.edu.tw (S.-M.H.); 8 Division of Cardiology and Cardiovascular Research Center, Department of Internal Medicine, Taipei Medical University Hospital, Taipei 11031, Taiwan; g870905@tmu.edu.tw; 9 Division of Nephrology, Department of Medicine, Tri-Service General Hospital, National Defense Medical Center, Taipei 11490, Taiwan; l521116@gmail.com; 10 Department of Molecular Microbiology and Immunology, Keck School of Medicine, University of Southern California, Los Angeles, CA 90033, USA; wanlin5@gmail.com; 11 Department of Physiology and Biophysics, Graduate Institute of Physiology, National Defense Medical Center, Taipei 11490, Taiwan

**Keywords:** carvedilol, atherosclerosis, cholesterol efflux, exosomes

## Abstract

Carvedilol (Cav), a nonselective β-blocker with α1 adrenoceptor blocking effect, has been used as a standard therapy for coronary artery disease. This study investigated the effects of Cav on exosome expression and function, ATP-binding cassette transporter A1 (ABCA1) expression, and cholesterol efflux that are relevant to the process of atherosclerosis. Human monocytic (THP-1) cell line and human hepatic (Huh-7) cells were treated with Cav, and cholesterol efflux was measured. Exosomes from cell culture medium or mice serum were isolated using glycan-coated recognition beads. Low-density lipoprotein receptor knockout (*ldlr*^−/−^) mice were fed with high-fat diet and treated with Cav. Cav accentuated cholesterol efflux and enhanced the expressions of ABCA1 protein and mRNA in both THP-1 and Huh-7 cells. In addition, Cav increased expression and function of exosomal ABCA1 in THP-1 macrophage exosomes. The mechanisms were associated with inhibition of nuclear factor-κB (NF-κB) and protein kinase B (Akt). In hypercholesterolemic *ldlr*^−/−^ mice, Cav enhanced serum exosomal ABCA1 expression and suppressed atherosclerosis by inhibiting lipid deposition and macrophage accumulation. Cav halts atherosclerosis by enhancing cholesterol efflux and increasing ABCA1 expression in macrophages and in exosomes, possibly through NF-κB and Akt signaling, which provides mechanistic insights regarding the beneficial effects of Cav on atherosclerotic cardiovascular disease.

## 1. Introduction

Beta-blockers have cardioprotective properties, decrease mortality in patients with coronary artery disease (CAD), and are the recommended therapy for CAD and congestive heart failure [1,2]. However, the cardioprotective ability of β-blockers varies considerably. Carvedilol (Cav), a nonselective β-blocker with α1 adrenoceptor blocking effects, has been shown to exert various pharmacological actions, such as antioxidant and anti-inflammation effects and reduction in vascular smooth muscle migration [3]. Cav and its metabolites prevent lipid peroxidation, such as oxidation of low-density lipoprotein (LDL) to oxidized LDL [4,5]. Moreover, it modulates cardiac inflammatory cytokines and fibrogenic cytokines, inhibits the expression of matrix metalloproteinase (MMP)-2 and MMP-9, and reduces tumor necrosis factor (TNF)-α-stimulated endothelial adhesiveness to human mononuclear cells [6,7,8]. Regarding the in vivo role in atherosclerosis, Cav has been shown to reduce the atherogenic process by suppressing proliferative and fatty lesions in hypercholesterolemic rabbits [9]. The in vitro and in vivo studies have demonstrated the multiple beneficial effects of Cav in the reduction of atherosclerosis.

Reverse cholesterol transport (RCT) is a critical process contributing to protection from atherosclerosis. The process involves transporting excess cellular cholesterol from the peripheral tissues to the liver for biliary secretion [10]. ATP-binding cassette transporter A1 (ABCA1) is the key reverse cholesterol transporter that mediates cholesterol exported from cells to Apolipoprotein(Apo)-A1 to synthesize high-density lipoprotein (HDL) [11]. Human epidemiology studies have shown an inverse relationship regarding HDL cholesterol and cardiovascular events [12,13].

Accumulating evidence suggests that exosomes have an influential role in intercellular communication in both normal and pathophysiological cardiovascular conditions [14]. Exosomes are functional vesicles that contain cell-specific proteins, lipids, and genetic materials and are considered to carry specific messages in proinflammatory conditions such as atherosclerosis [15]. For examples, under shear stress, exosomes derived from endothelial cells (ECs) present with miR-143/145, which control genes that prevent smooth muscle cells hyperplasia and support maintaining their contractile phenotype [16]. Exosomes released from ECs overexpressing Delta-like 4 factor (Dll-4) are taken up by the neighboring ECs. Transfer of these Exosomes through ECs can express Dll-4 that promotes the increase in angiogenesis by inhibiting Notch signaling [17]. Under exogenous stress stimuli, THP-1 derived exosomes containing miR-150 enter into human microvascular endothelial cells (HMVEC); application of exogenous miR-150 enhances migration of HMVECs through downregulation of c-Myb [18]. Moreover, exosomes from dendritic cells activate nuclear factor-κB (NF-κB) pathway and upregulate expression of proinflammatory molecules, including vascular cell adhesion molecule (VCAM), intercellular adhesion molecule (ICAM1), and E-selectin, which increase endothelial inflammation [19]. These results suggest the important role of exosomes that transfer bioactive cargos as intercellular communication in cardiovascular diseases.

In this study, we assessed the effects of Cav on ABCA1 expression and cholesterol efflux that are relevant to the process of atherosclerosis. Additionally, we investigated the role of macrophage-derived exosomes on ABCA1 functions and cholesterol efflux under Cav treatment. Based on the potential effects of Cav on the regulation of exosome functions and cholesterol efflux, we explored its effects on atherosclerosis in atherosclerosis-prone LDL receptor knockout (*ldlr^−/−^*) mice. 

## 2. Results

### 2.1. Treatment with Carvedilol Upregulated ABCA1 Expression and Cholesterol Efflux in Both Human Monocytic (THP-1) and Human Hepatic (Huh-7) Cells

**Figure 1:**
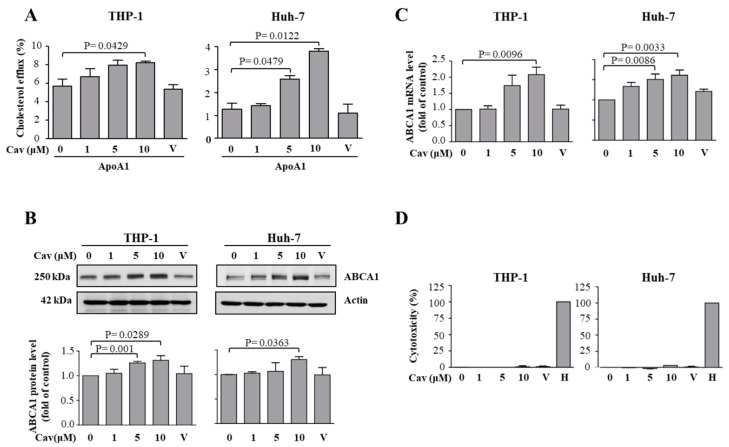
Carvedilol induced ABCA1 expression and enhanced cholesterol efflux in THP-1 macrophages and Huh-7 cells. (**A**) Total cholesterol efflux was evaluated after 24-h incubation of Apo-A1 or BSA in THP-1 macrophages or Huh-7 cells treated with various doses of Cav for 24 h. The average percentages of cholesterol efflux from at least three independent experiments are presented as the mean ± SEM (“V” indicates vehicle). (**B**) After treatment with various doses of Cav for 24 h, cell lysates were collected, and the expressions of ABCA1 and actin were determined. Representative data and relative protein expressions of ABCA1 in both THP-1 and Huh-7 cells are presented as the mean ± SEM after densitometric analysis. (**C**) The mRNA levels of ABCA1 and GAPDH were assessed by qRT-PCR and shown by the relative expressions. (**D**) THP-1 macrophages and Huh-7 cells were treated with Cav at the indicated concentrations for 24 h. Cell cytotoxicity was determined by LDH assay. “H” indicates high control of equal amounts of 1% Triton X-100-treated cells. (THP-1, human monocytic macrophage; Huh-7, human hepatic cell; Apo, apolipoprotein, BSA, bovine serum albumin; Cav, carvedilol; SEM, standard error of mean; ABCA1, ATP-binding cassette transport A1; GAPDH, Glyceraldehyde-3-Phosphate Dehydrogenase; qRT-PCR, quantitative reverse transcription polymerase chain reaction; LDH, lactate dehydrogenase).

To evaluate the effects of Cav on cholesterol efflux, human monocytic THP-1 or hepatic Huh-7 cells were treated with Cav. We found that treatment with Cav accentuated cholesterol efflux to Apo-A1 in both THP-1 and Huh-7 cells (Figure 1A). The ABCA1 protein levels significantly increased in Cav-treated THP-1 or Huh-7 cells (Figure 1B). Moreover, the mRNA levels of ABCA1 were significantly elevated in both cells (Figure 1C). As shown in Figure 1D, the concentrations of Cav used in the study have no apparent cytotoxicity or cell death effects on THP-1 or Huh-7 cells. Collectively, these results demonstrate that Cav increased ABCA1 expressions and its functions in both THP-1 and Huh-7 cells. Due to the critical role of macrophage regarding ABCA1 expression and cholesterol efflux capacity in the pathogenesis of atherosclerosis, we used THP-1 macrophages as our further in vitro model.

### 2.2. Increased ABCA1 Expression and Function in Exosomes of Carvedilol-Treated THP-1 Macrophages

**Figure 2:**
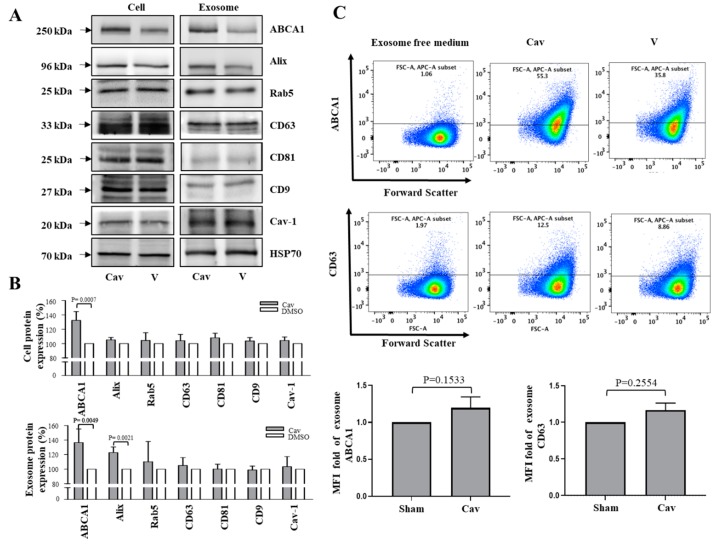
Carvedilol enhanced ABCA1 expressions on exosome in THP-1 macrophages. THP-1 macrophage treated with/without Cav. Cell lysates were collected and exosomal lysates were isolated using PEG. The expressions of ABCA1, and exosome specific biomarkers Alix, Rab5, CD63, CD81, CD9, Caveolin-1 and exosome internal control HSP70 were determined. Representative data (**A**) and relative protein expressions (**B**) in both THP-1 macrophage cell lysates and THP-1 macrophage exosomal lysates were presented as the mean ± SEM of three independent experiments in triplicate after densitometric analysis; (**C**) semiquantitative detection of THP-1 macrophage exosomes using glycan-coated recognition bead, EXÖBead^®^, to capture THP-1 macrophage exosomes in cell culture supernatants. EXÖBead^®^-exosome complexes were subsequently stained using anti-ABCA1, anti-CD63 antibody, and their secondary fluorescent antibody. Data were collected using a FACSCanto II (BD Biosciences, USA) flow cytometer and analyzed using FlowJo. Representative data of ABCA1/CD63 MFI from three independent experiments are presented. (PEG, polyethylene glycol; HSP70, heat shock protein 70; MFI, median fluorescence intensity).

Previous studies have suggested that exosomes are involved in the development of atherosclerosis; however, the exact mechanism is unknown [20]. To evaluate the effects of Cav on exosome biogenesis, cell culture medium (CCM) was collected from Cav or dimethyl sulfoxide (DMSO)-treated THP-1 macrophages, and exosomes were isolated from CCM by polyethylene glycol (PEG) base isolation and glycan-coated recognition bead EXÖBead^®^. Then, the effects of Cav on exosome biogenesis were evaluated, which showed that ABCA1 and the exosome biomarker Alix protein levels significantly increased in Cav-treated THP-1 macrophage exosomes (Figure 2A,B). To further understand the effects of Cav on exosomal ABCA1 expression, we evaluated ABCA1 expression on CD63-positive exosomes. By flow cytometry, treatment with Cav had a trend toward increasing exosomal ABCA1 and CD63 expression, although non-significantly (Figure 2C).

**Figure 3:**
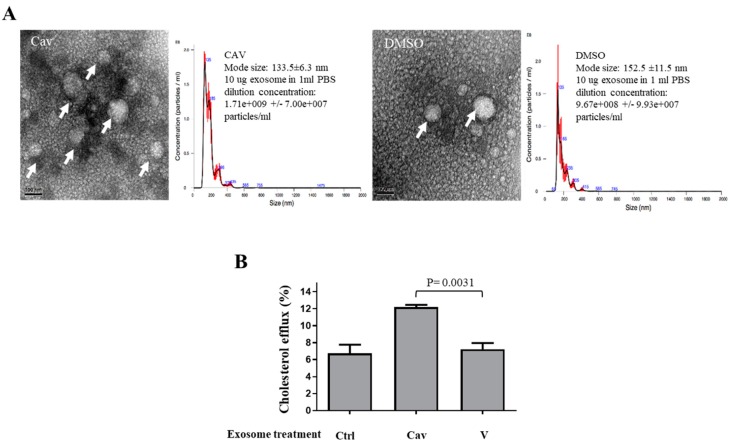
Carvedilol promotes exosomal functions of cholesterol efflux in THP-1 macrophages. (**A**) THP-1 macrophage exosomes were isolated using PEG and were observed through liquid-cell TEM. Size distribution of the particles that compose THP-1 macrophages exosomes, as measured by nanoparticle tracking analysis. (Arrow, exosome; concentration, mean values (black line) with error bars (red line)) (**B**) Exosomes were isolated from Cav (10 μM) or DMSO-treated THP-1 macrophages medium. Total cholesterol efflux was evaluated after 12-h incubation of ApoA1 or BSA in THP-1 macrophages treated with Cav/DMSO-treated THP-1 macrophages exosomes for 48 h. The average percentages of cholesterol efflux from at least three independent experiments are presented as the mean ± SEM. (TEM, transmission electron microscopy; DMSO, dimethyl sulfoxide; Ctrl, control).

Moreover, the numbers of Cav-treated THP-1 macrophage exosomes are higher than those of DMSO-treated control exosomes (Figure 3A). Then, we treated THP-1 macrophages with equal amounts of exosome from Cav or DMSO treated THP-1 macrophages, and showed that, compared with treatment with DMSO-treated exosomes, treatment with Cav-treated macrophage exosomes accentuated cholesterol efflux (Figure 3B). These findings provide evidence that Cav not only increase exosome number but also its function that helps cholesterol efflux.

### 2.3. Carvedilol Inhibited Akt and NF-κB Signaling in THP-1 Macrophages

**Figure 4:**
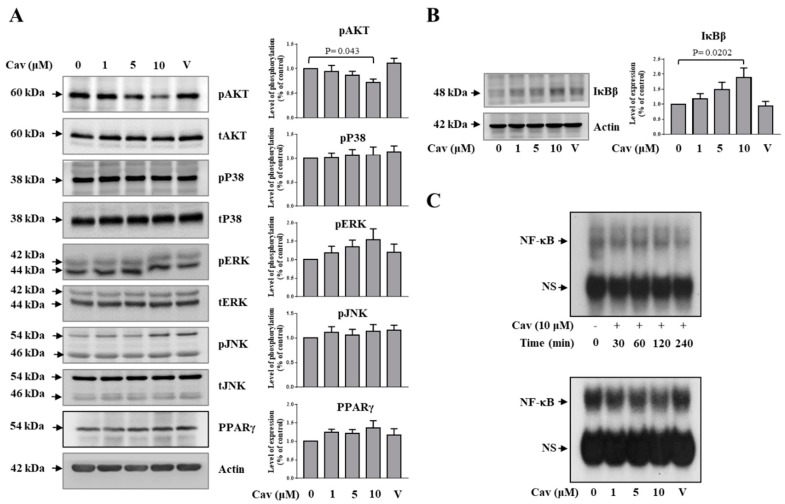
Carvedilol suppressed Akt and NF-κB signaling, but not MAPK signaling, in THP-1 macrophages. (**A**,**B**) THP-1 macrophages were treated with Cav at the indicated concentrations for 6 h. Cell lysates were collected and the expressions of pAkt, tAkt, pP38, tP38, pERK, tERK, pJNK, tJNK, PPAR-γ, IκBβ, and actin were determined. Representative data and relative protein expressions in THP-1 macrophages are presented as the mean ± SEM of three independent experiments in triplicate after densitometric analysis. (**C**) NF-κB DNA-binding activities in THP-1 macrophages treated with Cav (10 μM) at indicated time points or indicated doses of Cav at the time point of 6 h were analyzed by EMSA. Data shown are representative of at least three independent experiments. (Akt, protein kinase B; NF-κB, nuclear factor-κB; MAPK, mitogen-activated protein kinase; PPAR, peroxisome proliferator-activated receptor; IκB, inhibitor of kappa B; ERK, extracellular signal-regulated kinase; JNK, c-Jun N-terminal kinase; EMSA, electrophoresis mobility shift assay; NS, non-specific band).

To investigate the molecular mechanisms underlying the regulation of ABCA1 expression by Cav, we evaluated whether Cav regulates the NF-κB, protein kinase B (Akt), mitogen-activated protein kinase (MAPK), and peroxisome proliferator-activated receptors (PPAR)-γ signaling. We found that treatment with Cav reduced Akt, but not p-38, c-Jun N-terminal kinase (JNK), and extracellular signal-regulated kinase (ERK) activities at the time point of 6 h (Figure 4A). Moreover, treatment with Cav significantly increased inhibitor of kappa B (IκB) β expression (Figure 4B) and suppressed NF-κB DNA-binding activities (Figure 4C).

**Figure 5:**
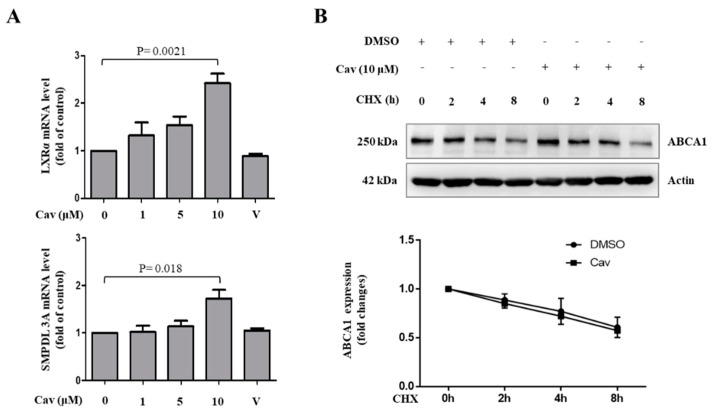
Carvedilol increased ABCA1 transcription, but not ABCA1 protein degradation, in THP-1 macrophages. (**A**) THP-1 macrophages were treated with various doses of Cav for 24 h. The mRNA levels of LXRα, SMPDL3A, and GAPDH were analyzed by qRT-PCR. The relative expressions of mRNA level are presented. (**B**) THP-1 cells were treated with or without Cav (10 μM) in the presence of cycloheximide (CHX, 2 μg/mL). Cell lysates were collected at the indicated time points, and the expressions of ABCA1 and actin were determined. The relative expression to each control group (with/without CHX) is presented. (LXR, liver X receptor; SMPDL3A, sphingomyelin phosphodiesterase acid like 3A).

Although no significant change was observed in the expression of PPAR-γ in Cav-treated THP-1 macrophages, we found that liver X receptor (LXR) and its downstream target gene, sphingomyelin phosphodiesterase acid like 3A (SMPDL3A), significantly increased in Cav-treated THP-1 macrophages at the concentration of 10 μM (Figure 5A). We further observed no significant effects of Cav on ABCA1 protein degradation (Figure 5B). These results demonstrated the potential regulatory mechanisms of ABCA1 expression in Cav-treated THP-1 macrophages.

### 2.4. Treatment with Carvedilol Inhibited Atherosclerosis Progression in Atherosclerosis-Prone ldlr^−/−^ Mice

**Figure 6:**
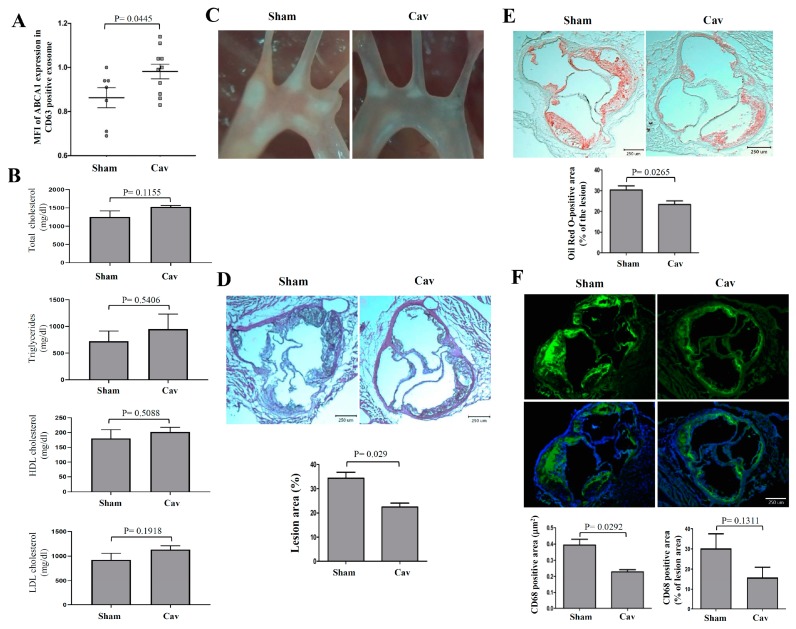
Cav inhibited the progression of atherosclerosis and reduced macrophage content. (**A**) Semiquantitative detection of mice serum exosomes using glycan-coated recognition bead, EXÖBead^®^, to capture exosomes from Cav-treated or sham group mice serum. EXÖBead^®^-exosome complexes were subsequently stained using anti-ABCA1, anti-CD63 antibody, and their secondary fluorescent antibody. The ratios of ABCA1/CD63 MFI are presented. (**B**) comparison of the lipid profiles, including total cholesterol, triglyceride, HDL cholesterol, and LDL cholesterol levels between Cav-treated (*n* = 6) and sham groups (*n* = 5); (**C**) representative light microscope pictures of the Cav-treated or sham-treated aortic arches (5× magnification); (**D**) representative histological analysis of cross-sections from the Cav-treated or sham-treated aortic sinus stained with hematoxylin and eosin (H&E), (**E**) Oil Red O staining, or (**F**) CD68 (green), and nuclear (blue) staining and quantification of the lesion area. All data are represented as mean ± SEM (*n* = 6–7 in each group).

To study the in vivo effects of Cav on atherosclerosis, we fed *ldlr^−/−^* mice a high fat diet (HFD) and treated them with Cav. We found that the ABCA1 expression on CD63 positive exosomes significantly increased in Cav-treated *ldlr^−/−^* mice compared with that in mice in the sham group (Figure 6A). However, the lipid profiles including total cholesterol, triglyceride, HDL cholesterol, and LDL cholesterol levels were not different significantly between Cav-treated and sham groups (Figure 6B). As shown in Figure 6C, light microscopy revealed markedly reduced atherosclerotic plaques in the aortic arch of Cav-treated *ldlr^−/−^* mice compared with that in mice in sham groups. The atherosclerotic lesion area in aortic sinus was significantly reduced in Cav-treated mice than in mice in sham groups (Figure 6D,E). Moreover, macrophage accumulation in aortic sinus significantly reduced in Cav-treated *ldlr^−/−^* mice compared with that in sham groups. While normalized with lesion area, treatment with Cav resulted in a trend toward reducing macrophage accumulation in atherosclerotic lesion of Cav-treated *ldlr^−/−^* mice (Figure 6F).

**Figure 7:**
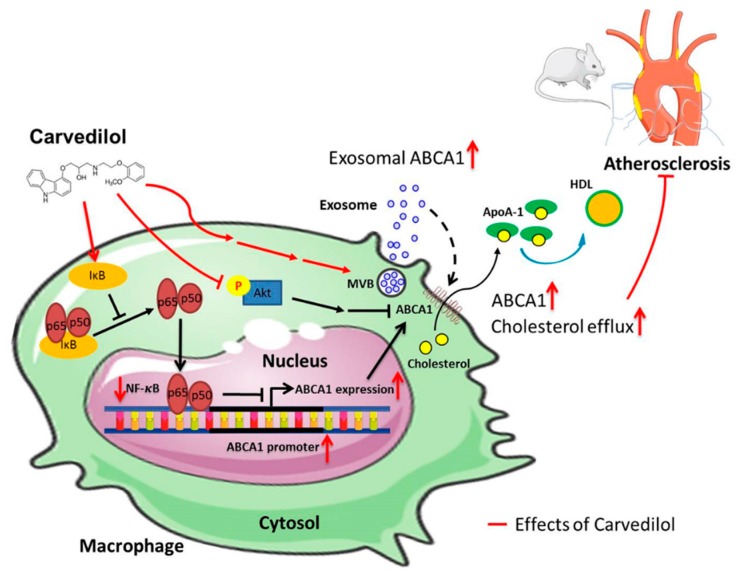
Proposed mechanisms of carvedilol on cholesterol efflux, exosome functions, and atherosclerosis. Cav enhanced cellular and exosomal ABCA1 expression and promoted cholesterol efflux in THP-1 macrophages, which contributes to the atheroprotective effects in mice model. Inhibition of NF-κB activation by enhancing IκB expression increases ABCA1-mediated cholesterol efflux. Inhibition of Akt increases cholesterol efflux to ApoA-1. These beneficial roles of Cav are possible through its suppression of NF-κB and Akt signaling. (Arrows, enhancement; T bars, inhibitory effect; dotted arrow, proposed enhancement effect).

Taken together, our results demonstrate that Cav significantly enhanced cholesterol efflux, increased ABCA1 expression and functions on exosomes, and halted the atherosclerotic progression of atherosclerosis-prone *ldlr^−/−^* mice, which may be attributed to the inhibition of Akt and NF-κB signaling. The working models of Cav on cholesterol efflux and atherosclerosis are shown in Figure 7.

## 3. Discussion

In this study, we demonstrated that Cav promotes cholesterol efflux in both THP-1 macrophages and Huh-7 cells. We found that Cav increased ABCA-1 expression, which might be associated with suppression of Akt and NF-κB signaling. Importantly, we first demonstrated that exosomes may possess therapeutic potential to deliver ABCA1 protein and promote cholesterol efflux. The molecular effects of Cav on cholesterol efflux and exosomal functions were further proven in atherosclerosis-prone *ldlr*^−/−^ mice. Our study provided the molecular mechanisms of the beneficial effects of Cav on atherosclerosis.

ABCA1 is highly expressed in macrophages where it promotes effluxed cellular lipids to assemble with lipid-poor pre-βHDL, forming nascent HDL particles [21]. Free cholesterol or cholesteryl esters in HDL may be directly cleared in the liver, through a process of selective uptake in which the lipid moiety of HDL is mostly removed and the protein portion is recycled into the circulation [22]. Mutations that inactivate ABCA1 lead to Tangier disease, a disorder characterized by increase extrahepatic tissue cholesterol accumulation, near-absence of plasma HDL, decrease plasma LDL concentrations, and increase plasma TG levels [23]. Therefore, macrophage ABCA1 plays a crucial role of initiation of reverse cholesterol transportation (RCT) at extrahepatic tissues. Liver is the major source of HDL biogenesis, whereas hepatocyte ABCA1 is responsible for the pool and recycling of plasma HDL by lipidating newly secreted apoA-I to produce pre-βHDL particles [22]. Over expression of hepatic ABCA1 can increase plasma HDL-C concentrations [24]. Hepatocyte-specific ABCA1 knock out mice shows marked reduction of plasma HDL-C due to defective assembly of nascent HDL by hepatocytes, reduction of plasma HDL-C resecretion from liver, and increase in trafficking of HDL-C into bile or feces [22,25]. However, deletion of hepatic specific ABCA1 in *ldlr*^−/−^ mice does not impair macrophage RCT and exacerbate atherogenesis [26]. These results underline the important role of hepatic ABCA1 regarding HDL synthesis and recycling, which mediate and maintain HDL catabolism. Our studies demonstrated that Cav enhance ABCA1 expression both on macrophages and Huh-7 cells, supporting its effects on modulating RCT, which mobilize excess oxLDL from endothelium and reduce vascular atherosclerosis.

Exosomes are bilayer membrane, endosome-derived, small extracellular vesicles that range from 30 to 150 nm in diameter. Genetic materials and lipids are actively and selectively incorporated into intraluminal vesicles, which reside within multivesicular endosomes and are the precursor of exosomes [27,28]. Exosomes have emerged as multifaceted regulators in a wide range of physiological and pathological cardiovascular processes, with the purpose of maintaining tissue homeostasis and coordinating the adaptive response to stress. Our study found that Alix protein levels significantly increased in exosomes from Cav-treated THP-1 macrophages compared with vehicle control, which demonstrated the modulation of exosome biogenesis in Cav-treated THP-1 macrophages and partly explained the increased number of exosomes in Cav-treated cells. Further siRNA studies knocking down the exosome biogenesis process are needed to verify its effects. Interestingly, ABCA1 activation during HDL biogenesis has been implicated in the regulation of microparticle formation, including that of exosomes. Such evidence suggests that exosome release is likely ABCA1 dependent and involves the regulation of cholesterol efflux [29,30]. Therefore, it is likely that Cav upregulates ABCA1 expression in macrophages and enhances the lipid particles carrying cargo of transmembrane proteins of RCTs into endosomes during the early formation of multivesicular bodies (MVBs) [31,32]. Accordingly, Cav enhances ABCA1 expression in macrophages, which might in turn upregulate the production of exosomes carrying ABCA1 proteins. Additional studies are necessary to explore the underlying mechanisms of exosomal ABCA1 regulation by Cav.

Cav-treated macrophage-derived exosomes considerably promote cholesterol efflux as well as enhance ABCA1 expression, suggesting that macrophage-derived exosomes are capable of transmitting signals that participate in the protective effects of Cav in atherosclerosis. The roles of exosome on atherosclerosis varies, depending on the origin of cells and pathophysiological conditions. Atherogenesis-prone adipose-derived exosomes exert proatherogenic effects by decreasing macrophages ABCA1 expression and cholesterol efflux [33]. Exosomes from macrophages suppress endothelial cell migration through control of integrin trafficking [16]. Foam cell-derived exosomes promote activation of Erk and Akt, and increase levels of vascular smooth muscle cells migration and adhesion [34]. Moreover, extracellular vesicle-derived miRNAs from atherogenic macrophages decrease the migratory capacity of naive recipient macrophages [32]. These results suggest the proatherogenic role of exosomes in pathological settings. However, endothelial cell-derived exosomes, under shear stress, can promote the de-differentiation and repress the proliferation of smooth muscle cells [35]. Our results showed that treatment with Cav increased exosomal ABCA1 expression both in cellular and murine models, suggesting that Cav may exert its antiatherogenic effect through exosome transmission. These findings indicate that exosome is a potential contributor to macrophage cholesterol homeostasis and is relevant in atherosclerosis. Exosome-mediated target therapy has therapeutic potential for modulation of the atherosclerotic process.

Cav suppresses NF-κB signaling by enhancing IκB expression and reducing NF-κB DNA-binding activities, suggesting its anti-inflammatory properties. NF-κB signaling was recognized to communicate inflammation and control multiple processes of atherosclerosis; however, its role in mediating cholesterol efflux remains unclear [36]. Consistent with our study, inhibition of NF-κB activation by using overexpression of IκBα in transgenic mice has been shown to reduce foam cell formation by controlling ABCA1-mediated cholesterol efflux [37]. Moreover, overexpression of macrophage-specific cholesteryl ester hydrolase, which generates free cholesterol for efflux pathway, attenuates NF-κB and AP-1 activation in transgenic mice and reduces atherosclerotic lesions [38]. However, under TNFα stimulation, mice macrophages show increased ABCA1 expression with activated NF-κB signaling, whereas IκB kinase beta-deficient macrophages show decreased ABCA1 expression [39]. These results suggest the dual function of NF-κB through activation of different signaling pathways in the regulation of ABCA1 in basal and proinflammatory status.

Phosphoinositide-3 kinase (PI3K) has been implicated in the control of macrophage cholesterol homeostasis. Dong et al. found that inhibition of Akt or mTORC1, which is a major downstream target of Akt, increases cholesterol efflux to ApoA-1 in RAW 264.7 macrophages, pancreatic beta cells, and hepatocytes [40]. Moreover, less cholesterol accumulation was observed in the macrophages of atherosclerosis-prone apolipoprotein E-deficient (*ApoE*^‒^/^‒)^ mice who underwent *Akt1*^‒^/^‒^ bone marrow transplantation when exposed to modified lipoproteins [41]. These results suggest that Akt may negatively regulate ABCA1 in macrophages, and that the antiatherogenic effect of Cav on macrophages may be partly associated with the Akt inhibition.

Our results show that Cav enhances ABCA1 mRNA expression and upregulates the expression of LXR. LXR is a nuclear receptor that functions as a cholesterol sensor regulating both cellular and systemic cholesterol homeostasis [42,43]. LXRs have also been shown to exert anti-inflammatory properties by suppressing genes involved in inflammation, such as inducible nitric-oxide synthase, NF-κB, and cyclooxygenase2 in murine macrophages [44]. Treatment of macrophages with LXRα activator increases the expression of ABCA1 mRNA and promotes cholesterol efflux to Apo-A1 [45]. The enhanced LXRα, SMPDL3A, and ABCA1 mRNA expression during Cav treatment as well as the absence of protective effects of Cav on ABCA1 protein degradation during cycloheximide treatment suggest that Cav regulates ABCA1 expression through the transcriptional level rather than protein stability.

Treatment with Cav does not significantly alter lipid profiles such as serum HDL, LDL, total cholesterol, and triglyceride levels in mice. The results are consistent with what was shown in earlier human studies that no significant changes in the serum HDL level in patients treated with Cav [46,47]. An experimental study of the oxidative stress on *ApoE*^‒^/^‒^ mice found no significant effects on serum total cholesterol and triglycerides after 8-week of Cav treatment [48]. Recent studies argued that the cholesterol efflux capacity is critical in atherosclerosis, a strong inverse association with coronary artery diseases that is independent of the circulatory HDL levels [12,13]. Therefore, we proposed that the beneficial effects of Cav in experimental atherosclerosis may in part be attributed to the improvement of HDL function and cellular cholesterol efflux, rather than HDL levels.

Although clinical studies have demonstrated the beneficial effects of Cav on the prevention and treatment of atherosclerotic cardiovascular disease, the underlying mechanisms that have been proposed in the literature mainly focus on its antioxidant effects, anti-inflammation effects, and the inhibition of the sympathetic system [3]. For example, free radicals and oxidative stress play critical roles in the initiation of atherosclerosis. Shimada et al. studied the effect of Cav on the severity of atherosclerosis in *ApoE*^‒^/^‒^ mice and found that the atheroprotective effects of Cav is likely mediated by the suppression of superoxide production [48]. The antioxidant activity of Cav is considered due to its chemical structure of carbazole moiety, which inhibits lipid peroxidation of cell membranes and reactive oxygen species (ROS) generation [49]. The antioxidative effects of Cav were shown in reduction of TNF-alpha stimulated intracellular ROS production, which activate redox sensitive nuclear factor kappa B and activator protein-1 transcription pathways that induced endothelial adhesiveness to human mononuclear cells [8]. Moreover, human peripheral blood T cells activation is inhibited by Cav treatment. The effect is through downregulating NF-kappaB activity, suggesting its antioxidative activity [50]. In addition to the molecular regulatory mechanisms of ABCA1 expression and cholesterol efflux, our study demonstrated that Cav regulates in vivo exosome functions and reduces lipid as well as macrophage accumulation in atherosclerotic plaques. These results further elucidate the protective effects of Cav on atherosclerosis.

This study has some limitations. First, we did not perform knockdown, inhibitor or stimulator experiments, the detailed regulatory mechanisms underlying the regulation of exosomal functions by Cav remain unclear. Second, we did not show the effects of Cav on in vivo cholesterol efflux capacity. Third, we did not evaluate the effects of Cav on the expressions of microRNAs in this study although ABCA1 is controlled by several microRNAs for posttranscriptional regulation. Fourth, we did not measure the physiological effects, such as blood pressure in Cav-treated mice. However, our study uncovers novel mechanisms underlying the effects of Cav on the reduction of atherosclerosis.

## 4. Materials and Methods

### 4.1. Chemicals

Cav was obtained from ApexBio (Cat. B1332; ApexBio Technology, Houston, TX, USA). ^3^H-labeled cholesterol was obtained from Perkin-Elmer Inc. (Boston, MA, USA). EXÖBead^®^ was purchased from Biovesicle Inc. (Taipei, Taiwan). Unless otherwise specified, all other reagents were purchased from Sigma-Aldrich Chemical Company (St. Louis, MO, USA).

### 4.2. Cell Culture

Human monocytic cell line (THP-1) and human hepatic (Huh-7) cells were procured from the Bioresource Collection and Research Center (Hsinchu, Taiwan) and American Type Tissue Collection, respectively. THP-1 cells were cultured in medium comprising RPMI 1640, 10% fetal bovine serum (FBS), streptomycin (100 µg/mL), and penicillin (100 U/mL). Cells were incubated with 100 ng/mL PMA for 3 days to differentiate into macrophages at a density of 1 × 10^6^ cells/mL. Huh-7 cells were grown in Dulbecco’s Modified Eagle Medium (DMEM) containing 10% FBS, streptomycin (100 µg/mL), and penicillin (100 U/mL) [51].

### 4.3. Measurement of Nonspecific Cytotoxicity of Cav

Nonspecific cytotoxicity of Cav was measured by lactate dehydrogenase (LDH)-releasing assay according to manufacturer instructions (Roche, Indianapolis, IN, USA). The cytotoxicity percentage was calculated as follows: ((sample value − medium control)/(positive control − medium control)) × 100. The sample values were the average absorbance values from triplicate measurements of the indicated doses of Cav-treated THP-1 macrophage supernatants after subtraction of absorbance values of background control. The averages of absorbance values from untreated cell culture supernatants were used as medium control. Equal amounts of 1% Triton X-100–treated cells were used as a positive control.

### 4.4. Cholesterol Efflux

Cells were incubated with 0.5 μCi/mL ^3^H-cholesterol (PerkinElmer) in 12-well plates (1 × 10^6^ cells/well) at 37 °C for 24 h. After washing with phosphate buffered saline (PBS), the cells were treated with indicated doses of Cav for 24 h in a medium with addition of 2% fatty acid-free BSA (FAFA, Sigma) and 2 μM acetyl-coenzyme A acetyltransferase inhibitor. To enhance the cholesterol efflux from cells, human ApoA1 (10 μg/mL) was added and incubated with the cells for another 24 h. Finally, the cells and the medium were collected, and radioactivity was measured [51]. The percentage of ^3^H-cholesterol in the media/total ^3^H-cholesterol content in the cell was calculated. The percentage of cholesterol efflux to ApoA1 by subtracting cholesterol efflux to BSA was presented.

### 4.5. Exosome Isolation by Polyethylene Glycol (PEG)

THP-1 cells were cultured in exosome free medium which contain 10% exosome free FBS [52]. After treatment with Cav/vehicle control, cell culture medium (CCM) was collected. To avoid cell debris and apoptosis body, CCM was pre-cleared by centrifugation at 350× *g* in 10 min and 2000× *g* in 10 min and then filtered with 0.22 μm filter. Ten ml pre-cleared CCM was incubated with 2 mL PEG overnight at 4 °C [53]. Exosomal pellets were collected by centrifugation 10,000× *g* at 4 °C in 20 min and then resuspended with 50 μL PBS for the following experiments.

### 4.6. Semiquantification Analysis of Cell Culture Medium (CCM) Exosomes or Mice Serum Exosomes by Glycan-Recognition Bead, EXÖBead^®^

Approximately 10 mL of precleared CCM or 80 µL of precleared mice serum was incubated with the glycan-base bead EXÖBead^®^ (Biovesicle Inc.) overnight at 4 °C [54,55]. Exosome free medium without culture cells was used as a negative control. The exosome-beads complex was washed twice in wash buffer (Biovesicle Inc.) at room temperature and incubated with 2.5 µg/mL of anti-ABCA1 or anti-CD63 antibody overnight at 4 °C. Finally, the exosome-beads complex was incubated with 5 µg/mL of Alexa Fluor^®^ 488-conjugated anti-mouse antibody. Antibody-stained exosome-beads complex was acquired using a BD™ Biosciences FACSCanto II flow cytometer (BD Biosciences, Franklin Lakes, NJ, USA). Data were analyzed using FlowJo software (Tree Star, Ashland, OR, USA).

### 4.7. High-Resolution Liquid-Cell Transmission Electron Microscopy (TEM)

High-resolution liquid-cell TEM was conducted by encapsulating a native THP-1 macrophages exosome between two conventional TEM grids coated with carbon layers suspended over holes [56]. THP-1 macrophage exosomes were placed directly on the bottom carbon-coated TEM grid (provided by the Center for Micro/Nano Science and Technology (CMNST), National Cheng Kung University, Taiwan) by the pipette and covered with the other grid. After preparing the liquid specimen, a conventional TEM microscope without modification of the TEM itself was used for such analysis. A JEOL JEM 2100 TEM (Tokyo, Japan) was used for in situ TEM image inspection, which was operated under an acceleration voltage of 200 kV.

### 4.8. Nanoparticle Tracking Analysis

Ten micrograms of exosome (measured through a Bradford assay) preparations were diluted in 1 mL of PBS. Samples were measured using NanoSight LM10 instrument (Malvern Instruments GmbH, Malvern, UK) with a 405-nm laser and a high-sensitivity scientific complementary metal–oxide–semiconductor (sCMOS) camera [57]. Every sample was individually injected into the NanoSight LM10 laser chamber by using a 1-mL syringe. Three 45-s recordings with a frame rate of 25 frames per second were fixed. Thirteen detection thresholds were taken for each sample. Mode and mean sizes of particles and concentration of particles were measured using NTA 3.1 software (Malvern Instruments, Malvern, UK).

### 4.9. Western Blotting

Enhanced chemiluminescence immunoblotting (Amersham, City, UK) was applied as described [51]. The Cav-treated cells were lysed in cell lysis buffer. Cell extracts were used to determine protein concentration. Equal quantities of whole cellular extracts were loaded into 8%–10% sodium dodecyl sulfate-polyacrylamide gels to separate proteins by electrophoresis. Once separated, the proteins were transferred to a nitrocellulose membrane. The membrane was blocked with tris buffered saline with tween 20 (TBS-T) and 5% nonfat milk for 1 h before overnight incubation at 4 °C with primary antibodies against total ERK, JNK, p38, peroxisome proliferator-activated receptor γ (PPARγ) (Santa Cruz Biotechnology, USA), actin (Chemicon, Temecula, CA, USA), and ABCA1, CD63, Alix and Rab5 (Abcam, Cambridge, UK), or phosphorylated ERK, JNK, p38, total Akt, phosphorylated Akt, phosphorylated IKB-β (Cell Signaling, Danvers, MA, USA), CD81, CD9, Heat Shock Protein 70 (HSP-70), and carveolin-1 (HansaBioMed Life Sciences, Tallinn, Estonia). Then, the membrane was washed with TBS-T buffer and incubated for 1 h with conjugated secondary antibodies (diluted 1:5000) in blocking buffer. Finally, the Western-Light chemiluminescent detection system was used for immunodetection.

### 4.10. Quantitative Reverse Transcription Polymerase Chain Reaction (qRT-PCR)

qRT-PCR analysis was performed by using SYBR Green Master Mix (Applied BioSystems, Foster City, CA, USA) following the manufacturer’s instructions [51]. After treatment with Cav, THP-1 cells were lysed with Trizol (Invitrogen, Carlsbad, CA, USA). The total RNAs were collected and then reverse transcribed to cDNAs. Each sample contains 10 ng of cDNA, 1× Master Mix, and gene-specific primers at a final concentration of 100 nM in a 20-μL total volume and was used for PCR. Reactions involved 40 cycles of denaturation at 95 °C, and annealing and extension at 60 °C, in an ABI 7300 real-time PCR system (Applied BioSystems, Foster City, CA, USA). The relative expression of gene was presented using the comparative CT method, also called the 2^−ΔΔCT^ method [58]. The primer sequences used in the study were as follows: GAPDH, 5′-ATGGGGAAGGTGAAGGTCG-3′ and 5′-TAAAAGCAGCCCTGGTGACC-3′; ABCA1, 5′-GGTGATGTTTCTGACCAATGTGA-3′ and 5′-TGTCCTCATACCAGTTGAGAGAC-3′; LXRα, 5′-AAGCCCTGCATGCCTACGT-3′ and 5′-TGCAGACGCAGTGCAAACA-3′; SMPDL3A, 5′-AGTAGCAAACCTCTGGAAAC-3′ and 5′-GTCAGTCTTGTTCAGTGTC-3′.

### 4.11. Nuclear Extract Preparation

Nuclear extraction procedure followed the protocol as described previously [59]. Cell pellet was suspended in 70 µL of buffer A (10 mM KCl; l0 mM HEPES, pH 7.9; 1.5 mM MgC1_2_; 1 mM PMSF; and 3.3 µg/mL aprotinin; 1 mM dithiothreitol (DTT)) with gentle vortexing at 4 °C for 15 min. Cell suspension was then centrifuged at 15,000 rpm for 3 min. Supernatants containing the cytoplasmic extract were removed. The collected pelleted nuclei were washed in 70 µL of Buffer A and centrifuged at 15,000 rpm for another 20 min. The collected nuclear pellets were resuspended in 25 µL of Buffer C (1.5 mM MgCl_2_; 420 mM NaCl; 20 mM HEPES, pH 7.9; 0.2 mM EDTA; 1 mM DTT; 0.5 mM PMSF; 25% glycerol; and 3.3 µg/mL aprotinin). The nuclear suspension was incubated for 20 min at 4 °C with occasional vigorous vortexing followed by centrifuged at 15,000 rpm for 20 min. Supernatants containing the nuclear extracts were collected for analysis.

### 4.12. Electrophoresis Mobility Shift Assay (EMSA)

DNA oligonucleotides containing the NF-κB binding site (5ʹ-AGTTGAGGGGACTTTCCCAGGC-3ʹ) were used as probes and were radiolabeled with [γ-^32^P] ATP using T4 kinase (Promega, Madison, WI, USA) [51]. Nuclear extracts were properly prepared and then mixed with the binding buffer (10 mM Tris-HCl, pH 7.5; 50 mM NaCl; 1 mM EDTA; 1 mM DTT; 5% glycerol; and 2 µg poly (dI-dC)) for 20 min incubation at 4 °C. Then, 5 µg of nuclear extraction buffer was incubated with the radiolabeled NF-κB probes for 20 min at room temperature. Next, the mixture was run in a 6.6% nondenaturing polyacrylamide gel with 0.5× Tris-borate/EDTA as the electrophoresis buffer at constant voltage. Finally, the gel was dried and exposed to X-ray film at −80 °C overnight.

### 4.13. Animals

The *ldlr*^−/−^ mice in C57BL/6 background were bred and maintained in pathogen-free conditions as per the protocol approved by the Animal Care and Use Committee of the National Defense Medical Center (IACUC-15-130, approved on 4 April 2015). The mice were fed a high-fat diet (HFD) and were randomly divided into two groups: HFD with Cav treatment and HFD without Cav treatment. At eight weeks of age, the mice were fed HFD for an additional 12 weeks. After feeding for 4 weeks, the mice underwent intervention treatment with Cav (10 mg/kg/day) or DMSO as the sham control by using an implantable ALZET^®^ osmotic pump model 2006 (Alza Co., Palo Alto, CA, USA). The osmotic pump has a pumping rate of 0.15 µL/h and reservoir volume of 200 µL, with duration of nearly 8 weeks [48]. Blood was collected by cardiac puncture method under anesthesia in mice aged 20 weeks, following which the mice were sacrificed. After PBS perfusion, the aorta and hearts were collected and stored in 4% paraformaldehyde for further staining.

### 4.14. Histology, Immunohistochemistry, and Plaque Analyses

The hearts were fixed in 4% paraformaldehyde for 24 h and then placed in 30% sucrose PBS buffer before being embedded at the optimal cutting temperature and frozen. Approximately 5-μm-thick sections were prepared for hematoxylin and eosin (H&E) staining and immunohistochemical analyses. For morphometric lesion analysis, the sections were stained with Mayer’s H&E (Atom Scientific, Manchester, UK). Sections for CD68 staining were fixed by immersion in ice-cold acetone/methanol (1:1) for 3 min. These sections were incubated in a blocking solution with 3% goat serum in PBS for 1 h at room temperature and then with monoclonal mouse anti-human CD68 antibody (Dako, Glostrup, Denmark) overnight. The sections were then incubated with a secondary antibody for 1 h at room temperature; subsequently, they were mounted on a mounting medium with DAPI for fluorescence (Vector Laboratories; Burlingame, CA, USA), and imaged using a fluorescence microscope (Leica, Wetzlar, Germany). The lesion area and CD68 positive area were measured by taking the average of three sections by using ImageJ 1.52 quantification (National Institutes of Health, Bethesda, MD, USA), as defined previously [60,61].

### 4.15. Oil-Red O Staining (ORO)

For the detection of neutral lipids, 10-μm-thick fresh frozen sections were rinsed with 78% methanol for 1 min and were then incubated with doubling dilutions of ORO working solution (Muto Pure Chemicals Co., Ltd., Bunkyo-ku, Japan) at room temperature for 50 min [62]. Then, the sections were incubated for 1 min each in separate solutions of 78% methanol, Mayer’s hemalum, and 0.05% lithium carbonate. They were rinsed under running tap water after each step, and then mounted after being dried. The lesion area was quantified as the percentage of ORO-positive staining area in the total lesion area.

### 4.16. Statistical Analysis

Comparison among multiple groups was conducted using Kruskal–Wallis test with Dunn’s post-hoc tests. Wilcoxon signed-rank test was used to compare between two groups. *p* < 0.05 was considered statistically significant.

## 5. Conclusions

Collectively, we demonstrated that Cav regulates ABCA1 expression and cholesterol efflux in both THP-1 and Huh-7 cells. Interestingly, for the first time, we provide evidence that exosomes can carry ABCA1 in macrophages, displaying their cholesterol efflux capacity. Besides exosomal transmission, the underlying regulatory mechanisms of Cav on ABCA1 expression are associated with suppression of NF-κB and Akt signaling. Additional in vivo and mechanistic studies are necessary to confirm the regulatory effects of Cav on exosome expressions and functions and the atherosclerosis process.

## Abbreviations

ABCA1	ATP-binding cassette transporter A1
Akt	Protein kinase B
AP-1	Activating protein-1
CAD	Coronary artery disease
Cav	Carvedilol
CCM	Cell culture medium
ECs	Endothelial cells
EMSA	Electrophoresis mobility shift assay
ERK	Extracellular signal-regulated kinase
HDL	High-density lipoprotein
Huh-7	Human hepatic cell line
IFN	Interferon
IκB	Inhibitor of kappa B
JNK	c-Jun N-terminal kinase
LDH	Lactate dehydrogenase
LDL	Low-density lipoprotein
Ldlr^−/−^	Low-density lipoprotein receptors knockout
LXR	Liver X receptor
MAPK	Mitogen-activated protein kinase
MTT	3-[4,5-Dimethylthiazol-2-yl]-2,5-diphenyl tetrazolium bromide
NF-κB	Nuclear factor-kappa B
ORO	Oil-red O staining
PPAR	Peroxisome proliferator-activated receptor
RCT	Reverse cholesterol transport
SMPDL3A	Sphingomyelin Phosphodiesterase Acid Like 3A
TEM	Transmission electron microscopy
THP-1	Human monocytic cell line
